# Bruceolline D: 3,3-dimethyl-1*H*,4*H*-cyclo­penta­[*b*]indol-2(3*H*)-one

**DOI:** 10.1107/S1600536813014955

**Published:** 2013-06-08

**Authors:** Justin M. Lopchuk, Gordon W. Gribble, Jerry P. Jasinski

**Affiliations:** a Department of Chemistry, Dartmouth College, Hanover, NH 03755-3564, USA; b Department of Chemistry, Keene State College, 229 Main Street, Keene, NH 03435-2001, USA

## Abstract

The title compound, C_13_H_13_NO, crystallizes with four independent mol­ecules in the asymmetric unit. The 12-membered penta­[*b*]indole rings are essentially planar, with maximum deviations ranging from 0.034 (4) to 0.036 (4) Å in the four unique mol­ecules. In the crystal, weak C—H⋯O inter­actions are observed, which link the mol­ecules into chains along [010].

## Related literature

For the first isolation of bruceolline D, see: Ouyang *et al.* (1994 ▶). For a Fischer indole synthesis approach to bruceolline D, see: Dashkevich (1978 ▶). For the methyl­ation of 2-methyl­cyclo­pentane-1,3-dione, see: Agosta & Smith (1970 ▶). For the palladium-catalysed synthesis of related fused indole structures, see: Nazare *et al.* (2004 ▶). For the isolation of related bruceollines, see: Chen *et al.* (2011 ▶). For the total synthesis and crystal structure of bruceolline E, see: Jordan *et al.* (2011 ▶, 2012 ▶). For standard bond lengths, see: Allen *et al.* (1987 ▶).
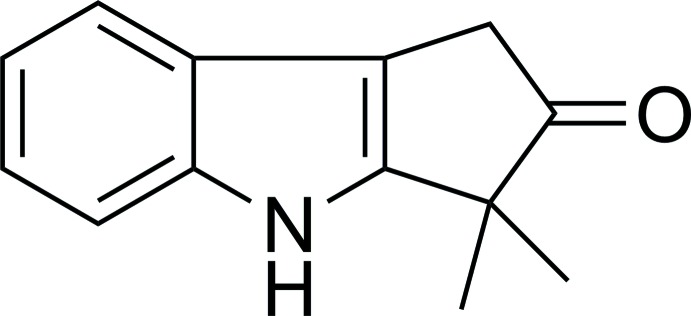


## Experimental

### 

#### Crystal data

C_13_H_13_NO
*M* *_r_* = 199.24
Orthorhombic, 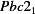

*a* = 10.13410 (14) Å
*b* = 21.9219 (3) Å
*c* = 19.3747 (3) Å
*V* = 4304.27 (11) Å^3^
*Z* = 16
Cu *K*α radiation
μ = 0.62 mm^−1^
*T* = 173 K
0.32 × 0.18 × 0.06 mm

#### Data collection

Agilent Xcalibur (Eos, Gemini) diffractometer
Absorption correction: multi-scan (*CrysAlis PRO* and *CrysAlis RED*; Agilent, 2012 ▶) *T*_min_ = 0.876, *T*_max_ = 1.000
28235 measured reflections
7612 independent reflections
7097 reflections with *I* > 2σ(*I*)
*R*_int_ = 0.038

#### Refinement

*R*[*F* ^2^ > 2σ(*F* ^2^)] = 0.068
*wR*(*F* ^2^) = 0.174
*S* = 1.04
7612 reflections
549 parameters
1 restraint
H-atom parameters constrained
Δρ_max_ = 0.69 e Å^−3^
Δρ_min_ = −0.33 e Å^−3^

### 

Data collection: *CrysAlis PRO* (Agilent, 2012 ▶); cell refinement: *CrysAlis PRO*; data reduction: *CrysAlis PRO*; program(s) used to solve structure: *SUPERFLIP* (Palatinus & Chapuis, 2007 ▶); program(s) used to refine structure: *SHELXL2012* (Sheldrick, 2008 ▶); molecular graphics: *OLEX2* (Dolomanov *et al.*, 2009 ▶); software used to prepare material for publication: *OLEX2*.

## Supplementary Material

Crystal structure: contains datablock(s) global, I. DOI: 10.1107/S1600536813014955/sj5328sup1.cif

Structure factors: contains datablock(s) I. DOI: 10.1107/S1600536813014955/sj5328Isup2.hkl

Supplementary material file. DOI: 10.1107/S1600536813014955/sj5328Isup3.cml

Additional supplementary materials:  crystallographic information; 3D view; checkCIF report

## Figures and Tables

**Table 1:** Hydrogen-bond geometry (Å, °)

*D*—H⋯*A*	*D*—H	H⋯*A*	*D*⋯*A*	*D*—H⋯*A*
C9*B*—H9*B*⋯O1*B* ^i^	0.95	2.51	3.427 (4)	162
C10*B*—H10*B*⋯O1*A*	0.95	2.61	3.379 (5)	139
C12*B*—H12*D*⋯O1*C* ^ii^	0.98	2.73	3.497 (6)	136
C9*D*—H9*D*⋯O1*D* ^iii^	0.95	2.46	3.372 (4)	161
C10*D*—H10*D*⋯O1*C* ^ii^	0.95	2.46	3.274 (5)	144
C12*D*—H12*J*⋯O1*A* ^iv^	0.98	2.69	3.485 (6)	138

Symmetry codes: (i) 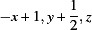; (ii) 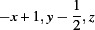; (iii) 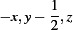; (iv) 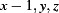.

**Fig. 1.**
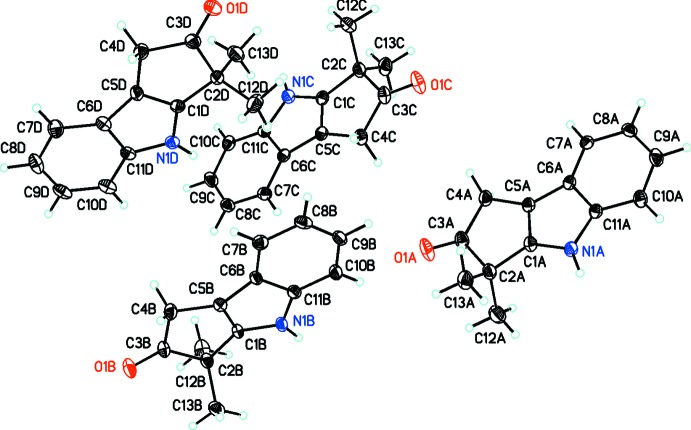
Molecular structure of the title compound showing the atom labeling scheme and 30% probability displacement ellipsoids.

**Fig. 2.**
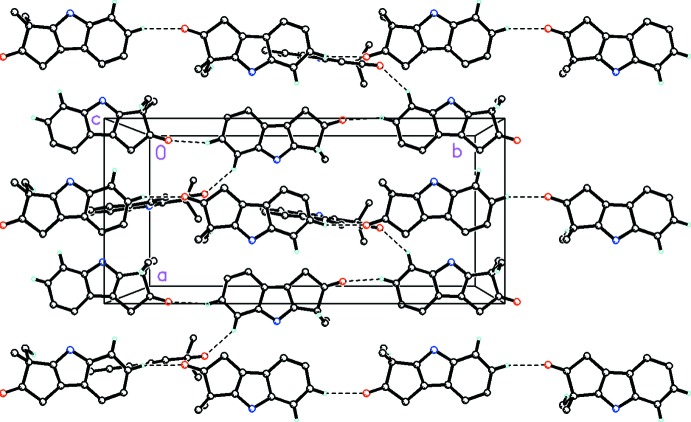
Packing diagram of the title compound viewed along the c axis. Dashed lines indicate weak C—H···O intermolecular interactions forming 1-D chains along (010). H atoms not involved in these intermolecular interactions have been omitted for clarity.

**Fig. 3.**
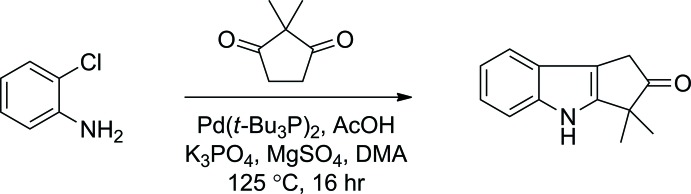
Synthesis of (I).

## supplementary crystallographic information

### Comment

Bruceolline D is a cyclopent[*b*]indole alkaloid which was first isolated from the root wood of *Brucea mollis* var. *tonkinensis* (Ouyang *et al.*, 1994). Our total synthesis of bruceolline D was achieved by methylation of 2-methylcyclopentane-1,3-dione (Agosta *et al.*, 1970) followed by a palladium-catalyzed cyclization with 2-chloroaniline in 88% yield (Nazare *et al.*, 2004). The reaction of phenylhydrazine with 2-methylcyclopentane-1,3-dione (Fischer indole synthesis) under thermal and acidic conditions has been investigated (Dashkevich, 1978). Other structurally similar bruceollines have been isolated more recently (Chen *et al.*, 2011). Bruceolline E has been synthesized by a sequential Nazarov cyclization/selenium dioxide oxidation (Jordan *et al.*, 2011) and the crystal structure determined (Jordan *et al.*, 2012). In view of the importance of cyclopent[*b*]indole alkaloids, we report here the crystal structure of the title compound, C_13_H_13_NO, (I).

The title compound, (I), crystallizes with four molecules in the asymmetric unit (Fig. 1). In the planar 12-member cyclopenta[*b*]indol-2(1*H*,3*H*,4*H*) rings, the maximum deviations from planarity are at C4A, 0.034 (4) Å, C3B, 0.034 (4) Å, C4C, -0.036 (4) Å and C4D, -0.035 (4) Å) atoms, respectively. Bond lengths are in normal ranges (Allen *et al.*, 1987). In the crystal weak intermolecular C—H···O interactions are observed (Table 1) which link the molecules into chains along [010] and contribute to crystal packing stability (Fig. 2).

### Experimental

2-Chloroaniline (255 mg, 2.0 mmol, 1 equiv.), 2,2-dimethylcyclopentane-1, 3-dione (757 mg, 6.0 mmol, 3 equiv.), acetic acid (180 mg, 3.0 mmol, 3 equiv.), magnesium sulfate (120 mg, 1.0 mmol, 0.5 equiv.), and dimethylacetamide (6 mL) were added to a 25 mL round bottom flask. After bubbling argon through the mixture for 10 minutes, potassium phosphate tribasic (552 mg, 2.6 mmol, 1.3 equiv.) and Pd(*t*-Bu_3_P)_2_ (101 mg, 0.2 mmol, 0.1 equiv.) were added and the flask sealed with a septum. After bubbling argon through the reaction mixture for an additional 5 minutes, the flask was heated for 16 hours at 125°C. After the reaction was complete (monitored by TLC), the mixture was cooled to room temperature and filtered to remove insoluble material (Fig. 3). The filtered solids were washed with dimethylacetamide (3 x 2 mL). Water (40 mL) was added to the filtrate and the aqueous mixture was extracted with ethyl acetate (3 x 50 mL). The combined organic layers were dried over sodium sulfate, filtered, and concentrated *in vacuo*. The residue was purified by flash chromatography (20% ethyl acetate in pentane) to yield the desired product (I) as a pale yellow solid (350 mg, 88%). Single crystals suitable for diffraction were grown from dichloromethane layered with pentane (liquid/liquid diffusion) at ambient temperature [m.p. 434–436 K (dec); literature value 433–435 K (dec) (Ouyang *et al.*, (1994)].

### Refinement

All of the H atoms were placed in their calculated positions and refined using a riding model with Atom—H lengths of 0.95Å (CH), 0.99Å (CH_2_), 0.98Å (CH_3_) or 0.88Å (NH). Isotropic displacement parameters for these atoms were set to 1.2 (CH, CH_2_ NH) or 1.5 (CH_3_) times *U*_eq_ of the parent atom. All methyl substituents were refined as rotating groups. The maximum and minimum residual electron density peaks of 0.69 and -0.33 eÅ^-3^ were located 1.38 Å, and 1.31 Å from the H12D, and C11D atoms, respectively.

### Crystal data

**Table:**

C_13_H_13_NO	*D*_x_ = 1.230 Mg m^−^^3^
*M**_r_* = 199.24	Cu *K*α radiation, λ = 1.5418 Å
Orthorhombic, *P**b**c*2_1_	Cell parameters from 11183 reflections
*a* = 10.13410 (14) Å	θ = 3.0–72.4°
*b* = 21.9219 (3) Å	µ = 0.62 mm^−^^1^
*c* = 19.3747 (3) Å	*T* = 173 K
*V* = 4304.27 (11) Å^3^	Prism, colourless
*Z* = 16	0.32 × 0.18 × 0.06 mm
*F*(000) = 1696	

### Data collection

**Table:**

Agilent Xcalibur (Eos, Gemini) diffractometer	7612 independent reflections
Radiation source: Enhance (Cu) X-ray Source	7097 reflections with *I* > 2σ(*I*)
Graphite monochromator	*R*_int_ = 0.038
Detector resolution: 16.0416 pixels mm^-1^	θ_max_ = 72.6°, θ_min_ = 4.0°
ω scans	*h* = −12→12
Absorption correction: multi-scan (*CrysAlis PRO* and *CrysAlis RED*; Agilent, 2012)	*k* = −26→19
*T*_min_ = 0.876, *T*_max_ = 1.000	*l* = −20→23
28235 measured reflections	

### Refinement

**Table:**

Refinement on *F*^2^	1 restraint
Least-squares matrix: full	Hydrogen site location: inferred from neighbouring sites
*R*[*F*^2^ > 2σ(*F*^2^)] = 0.068	H-atom parameters constrained
*wR*(*F*^2^) = 0.174	*w* = 1/[σ^2^(*F*_o_^2^) + (0.1346*P*)^2^ + 0.5786*P*] where *P* = (*F*_o_^2^ + 2*F*_c_^2^)/3
*S* = 1.04	(Δ/σ)_max_ < 0.001
7612 reflections	Δρ_max_ = 0.69 e Å^−^^3^
549 parameters	Δρ_min_ = −0.33 e Å^−^^3^

### Special details

**Table:**

Geometry. All esds (except the esd in the dihedral angle between two l.s. planes) are estimated using the full covariance matrix. The cell esds are taken into account individually in the estimation of esds in distances, angles and torsion angles; correlations between esds in cell parameters are only used when they are defined by crystal symmetry. An approximate (isotropic) treatment of cell esds is used for estimating esds involving l.s. planes.

### Fractional atomic coordinates and isotropic or equivalent isotropic displacement parameters (Å^2^)

**Table:**

	*x*	*y*	*z*	*U*_iso_*/*U*_eq_	
O1A	0.9027 (4)	0.55207 (13)	0.52430 (17)	0.0587 (9)	
N1A	0.9928 (3)	0.72326 (13)	0.67458 (17)	0.0311 (7)	
H1A	1.0014	0.7162	0.7191	0.037*	
C1A	0.9695 (3)	0.68058 (15)	0.62468 (19)	0.0301 (7)	
C2A	0.9463 (4)	0.61292 (16)	0.6263 (2)	0.0353 (8)	
C3A	0.9229 (4)	0.60183 (16)	0.5480 (2)	0.0384 (8)	
C4A	0.9289 (4)	0.66154 (16)	0.50654 (19)	0.0353 (8)	
H4AA	0.9983	0.6600	0.4707	0.042*	
H4AB	0.8430	0.6709	0.4846	0.042*	
C5A	0.9618 (3)	0.70689 (15)	0.56179 (18)	0.0279 (6)	
C6A	0.9823 (3)	0.77087 (15)	0.57072 (19)	0.0268 (7)	
C7A	0.9849 (3)	0.82163 (16)	0.5269 (2)	0.0301 (7)	
H7A	0.9746	0.8169	0.4785	0.036*	
C8A	1.0027 (3)	0.87872 (16)	0.5556 (2)	0.0347 (8)	
H8A	1.0037	0.9135	0.5264	0.042*	
C9A	1.0194 (4)	0.88624 (17)	0.6267 (2)	0.0382 (9)	
H9A	1.0310	0.9262	0.6447	0.046*	
C10A	1.0195 (4)	0.83725 (16)	0.6713 (2)	0.0339 (8)	
H10A	1.0321	0.8427	0.7195	0.041*	
C11A	1.0003 (3)	0.77912 (15)	0.64301 (19)	0.0271 (7)	
C12A	1.0664 (4)	0.57622 (19)	0.6513 (3)	0.0545 (11)	
H12A	1.1450	0.5890	0.6255	0.082*	
H12B	1.0507	0.5326	0.6436	0.082*	
H12C	1.0802	0.5837	0.7006	0.082*	
C13A	0.8226 (4)	0.59501 (19)	0.6668 (2)	0.0455 (9)	
H13A	0.8369	0.6027	0.7160	0.068*	
H13B	0.8041	0.5516	0.6596	0.068*	
H13C	0.7474	0.6193	0.6506	0.068*	
O1B	0.4157 (3)	0.15987 (11)	0.55334 (18)	0.0456 (7)	
N1B	0.6744 (3)	0.34522 (12)	0.56110 (16)	0.0312 (6)	
H1B	0.7609	0.3419	0.5635	0.037*	
C1B	0.5864 (3)	0.29805 (14)	0.55585 (18)	0.0273 (6)	
C2B	0.6016 (3)	0.23033 (14)	0.5527 (2)	0.0312 (7)	
C3B	0.4528 (3)	0.21198 (15)	0.5510 (2)	0.0323 (7)	
C4B	0.3632 (3)	0.26770 (14)	0.5476 (2)	0.0339 (7)	
H4BA	0.3148	0.2695	0.5032	0.041*	
H4BB	0.2993	0.2682	0.5862	0.041*	
C5B	0.4611 (3)	0.31874 (15)	0.55362 (19)	0.0289 (7)	
C6B	0.4668 (3)	0.38412 (14)	0.55765 (18)	0.0282 (7)	
C7B	0.3743 (3)	0.43185 (16)	0.55652 (18)	0.0339 (7)	
H7B	0.2825	0.4235	0.5541	0.041*	
C8B	0.4203 (4)	0.49188 (15)	0.55907 (19)	0.0366 (8)	
H8B	0.3590	0.5246	0.5583	0.044*	
C9B	0.5547 (4)	0.50435 (16)	0.5627 (2)	0.0368 (8)	
H9B	0.5828	0.5457	0.5641	0.044*	
C10B	0.6479 (4)	0.45913 (15)	0.56428 (19)	0.0345 (7)	
H10B	0.7394	0.4684	0.5669	0.041*	
C11B	0.6032 (3)	0.39884 (14)	0.56189 (18)	0.0295 (7)	
C12B	0.6717 (4)	0.20825 (18)	0.4870 (2)	0.0469 (10)	
H12D	0.6260	0.2245	0.4464	0.070*	
H12E	0.6705	0.1636	0.4855	0.070*	
H12F	0.7632	0.2226	0.4872	0.070*	
C13B	0.6669 (4)	0.20277 (16)	0.6170 (2)	0.0446 (9)	
H13D	0.7598	0.2151	0.6187	0.067*	
H13E	0.6613	0.1582	0.6148	0.067*	
H13F	0.6214	0.2174	0.6584	0.067*	
O1C	0.6041 (4)	0.69989 (13)	0.37896 (17)	0.0555 (8)	
N1C	0.5290 (3)	0.52512 (13)	0.23148 (17)	0.0328 (7)	
H1C	0.5229	0.5313	0.1867	0.039*	
C1C	0.5509 (3)	0.56889 (15)	0.28084 (18)	0.0294 (7)	
C2C	0.5704 (4)	0.63639 (16)	0.27780 (19)	0.0347 (7)	
C3C	0.5900 (3)	0.64918 (16)	0.3560 (2)	0.0365 (8)	
C4C	0.5840 (4)	0.59052 (16)	0.39915 (19)	0.0349 (7)	
H4CA	0.6690	0.5825	0.4226	0.042*	
H4CB	0.5126	0.5923	0.4339	0.042*	
C5C	0.5558 (3)	0.54359 (15)	0.34498 (18)	0.0288 (6)	
C6C	0.5340 (3)	0.47964 (14)	0.33686 (18)	0.0267 (7)	
C7C	0.5275 (3)	0.42955 (16)	0.3818 (2)	0.0318 (7)	
H7C	0.5378	0.4352	0.4301	0.038*	
C8C	0.5058 (3)	0.37180 (16)	0.3550 (2)	0.0348 (8)	
H8C	0.5002	0.3377	0.3850	0.042*	
C9C	0.4921 (4)	0.36376 (16)	0.2839 (2)	0.0372 (9)	
H9C	0.4776	0.3238	0.2666	0.045*	
C10C	0.4987 (3)	0.41164 (16)	0.2377 (2)	0.0334 (8)	
H10C	0.4904	0.4053	0.1894	0.040*	
C11C	0.5181 (3)	0.46927 (15)	0.2650 (2)	0.0280 (7)	
C12C	0.4486 (4)	0.67119 (17)	0.2516 (3)	0.0506 (11)	
H12G	0.4339	0.6615	0.2028	0.076*	
H12H	0.4632	0.7151	0.2568	0.076*	
H12I	0.3711	0.6591	0.2786	0.076*	
C13C	0.6936 (4)	0.6559 (2)	0.2375 (2)	0.0460 (9)	
H13G	0.7707	0.6341	0.2555	0.069*	
H13H	0.7068	0.6999	0.2428	0.069*	
H13I	0.6819	0.6460	0.1886	0.069*	
O1D	−0.0630 (3)	0.59408 (11)	0.35172 (17)	0.0451 (7)	
N1D	0.1833 (3)	0.40513 (11)	0.35371 (16)	0.0299 (6)	
H1D	0.2700	0.4070	0.3551	0.036*	
C1D	0.0984 (3)	0.45404 (15)	0.35461 (18)	0.0282 (6)	
C2D	0.1177 (3)	0.52123 (14)	0.35591 (19)	0.0307 (7)	
C3D	−0.0296 (3)	0.54136 (15)	0.3543 (2)	0.0333 (8)	
C4D	−0.1232 (3)	0.48702 (16)	0.3542 (2)	0.0375 (8)	
H4DA	−0.1779	0.4860	0.3965	0.045*	
H4DB	−0.1815	0.4874	0.3132	0.045*	
C5D	−0.0288 (3)	0.43463 (15)	0.3519 (2)	0.0307 (7)	
C6D	−0.0265 (3)	0.36943 (15)	0.3490 (2)	0.0315 (7)	
C7D	−0.1231 (4)	0.32326 (17)	0.3459 (2)	0.0399 (8)	
H7D	−0.2143	0.3331	0.3444	0.048*	
C8D	−0.0809 (4)	0.26286 (17)	0.3451 (2)	0.0430 (9)	
H8D	−0.1447	0.2311	0.3434	0.052*	
C9D	0.0526 (4)	0.24775 (16)	0.3467 (2)	0.0394 (8)	
H9D	0.0779	0.2060	0.3463	0.047*	
C10D	0.1482 (4)	0.29203 (15)	0.34891 (19)	0.0372 (8)	
H10D	0.2391	0.2815	0.3495	0.045*	
C11D	0.1085 (3)	0.35272 (14)	0.35022 (18)	0.0304 (7)	
C12D	0.1847 (4)	0.5439 (2)	0.4218 (2)	0.0487 (10)	
H12J	0.1354	0.5294	0.4621	0.073*	
H12K	0.1864	0.5886	0.4218	0.073*	
H12L	0.2752	0.5283	0.4237	0.073*	
C13D	0.1868 (4)	0.54571 (17)	0.2920 (2)	0.0469 (10)	
H13J	0.2803	0.5344	0.2934	0.070*	
H13K	0.1789	0.5902	0.2907	0.070*	
H13L	0.1459	0.5283	0.2506	0.070*	

### Atomic displacement parameters (Å^2^)

**Table:**

	*U*^11^	*U*^22^	*U*^33^	*U*^12^	*U*^13^	*U*^23^
O1A	0.093 (3)	0.0354 (15)	0.0473 (17)	−0.0261 (15)	0.0177 (17)	−0.0125 (12)
N1A	0.0434 (16)	0.0309 (15)	0.0191 (15)	−0.0056 (11)	0.0003 (11)	−0.0026 (11)
C1A	0.0331 (16)	0.0282 (16)	0.0289 (18)	−0.0043 (12)	0.0039 (13)	−0.0030 (13)
C2A	0.0396 (18)	0.0309 (17)	0.036 (2)	−0.0089 (14)	0.0013 (15)	0.0004 (14)
C3A	0.0433 (19)	0.0360 (18)	0.0359 (19)	−0.0109 (14)	0.0103 (15)	−0.0050 (15)
C4A	0.0418 (18)	0.0346 (18)	0.0296 (18)	−0.0074 (14)	−0.0006 (15)	−0.0044 (14)
C5A	0.0297 (14)	0.0294 (15)	0.0246 (17)	−0.0031 (12)	0.0031 (12)	−0.0038 (13)
C6A	0.0249 (13)	0.0302 (17)	0.0253 (17)	−0.0019 (11)	0.0011 (12)	−0.0030 (13)
C7A	0.0284 (15)	0.0322 (17)	0.0298 (19)	−0.0015 (12)	−0.0036 (13)	0.0016 (14)
C8A	0.0301 (15)	0.0266 (16)	0.047 (3)	0.0029 (12)	−0.0020 (15)	0.0054 (17)
C9A	0.0363 (18)	0.0285 (17)	0.050 (3)	0.0012 (13)	0.0013 (16)	−0.0117 (17)
C10A	0.0384 (18)	0.0317 (18)	0.032 (2)	−0.0030 (13)	0.0054 (15)	−0.0081 (15)
C11A	0.0286 (15)	0.0296 (17)	0.0231 (19)	−0.0011 (12)	0.0020 (12)	−0.0041 (13)
C12A	0.048 (2)	0.037 (2)	0.079 (3)	−0.0004 (17)	−0.006 (2)	0.005 (2)
C13A	0.051 (2)	0.049 (2)	0.036 (2)	−0.0195 (17)	0.0049 (17)	0.0028 (16)
O1B	0.0417 (14)	0.0260 (12)	0.069 (2)	−0.0092 (10)	0.0051 (14)	−0.0036 (12)
N1B	0.0253 (12)	0.0252 (12)	0.0432 (17)	−0.0049 (9)	−0.0014 (12)	0.0024 (11)
C1B	0.0288 (14)	0.0213 (14)	0.0319 (17)	−0.0054 (11)	0.0013 (13)	−0.0010 (12)
C2B	0.0284 (15)	0.0244 (15)	0.041 (2)	−0.0005 (11)	0.0026 (14)	−0.0028 (13)
C3B	0.0289 (15)	0.0291 (16)	0.039 (2)	−0.0075 (12)	0.0052 (14)	−0.0022 (14)
C4B	0.0246 (14)	0.0309 (16)	0.046 (2)	−0.0035 (12)	0.0009 (14)	−0.0026 (14)
C5B	0.0296 (15)	0.0256 (15)	0.0316 (19)	−0.0002 (12)	−0.0010 (13)	0.0010 (13)
C6B	0.0378 (17)	0.0210 (15)	0.0259 (18)	−0.0045 (11)	−0.0021 (13)	0.0002 (12)
C7B	0.0355 (16)	0.0349 (17)	0.0312 (17)	0.0017 (13)	−0.0011 (14)	−0.0003 (14)
C8B	0.054 (2)	0.0264 (16)	0.0290 (18)	0.0120 (14)	−0.0023 (16)	0.0009 (14)
C9B	0.059 (2)	0.0238 (16)	0.0274 (18)	−0.0080 (14)	−0.0063 (16)	0.0031 (13)
C10B	0.0397 (17)	0.0307 (16)	0.0331 (18)	−0.0095 (13)	−0.0014 (14)	0.0028 (14)
C11B	0.0331 (15)	0.0270 (15)	0.0285 (17)	0.0001 (12)	−0.0003 (13)	0.0003 (13)
C12B	0.039 (2)	0.042 (2)	0.060 (3)	−0.0008 (15)	0.0147 (18)	−0.0147 (18)
C13B	0.041 (2)	0.0298 (18)	0.063 (3)	0.0036 (14)	−0.0026 (18)	0.0105 (17)
O1C	0.080 (2)	0.0346 (14)	0.0520 (18)	−0.0222 (14)	0.0161 (16)	−0.0098 (12)
N1C	0.0434 (16)	0.0320 (16)	0.0229 (16)	−0.0052 (11)	−0.0011 (12)	−0.0013 (12)
C1C	0.0256 (15)	0.0336 (16)	0.0290 (18)	−0.0027 (12)	0.0017 (13)	−0.0030 (13)
C2C	0.0385 (17)	0.0318 (17)	0.0339 (19)	−0.0077 (13)	0.0003 (14)	0.0007 (14)
C3C	0.0373 (17)	0.0343 (18)	0.038 (2)	−0.0114 (13)	0.0063 (15)	−0.0059 (15)
C4C	0.0399 (18)	0.0328 (17)	0.0320 (18)	−0.0050 (13)	0.0017 (15)	−0.0045 (14)
C5C	0.0238 (13)	0.0320 (15)	0.0305 (17)	−0.0016 (11)	0.0006 (13)	−0.0021 (13)
C6C	0.0230 (13)	0.0305 (16)	0.0264 (18)	0.0009 (11)	−0.0003 (12)	−0.0022 (13)
C7C	0.0309 (15)	0.0314 (17)	0.033 (2)	0.0024 (13)	−0.0005 (13)	0.0034 (14)
C8C	0.0300 (15)	0.0283 (17)	0.046 (2)	0.0033 (12)	0.0000 (15)	0.0055 (17)
C9C	0.0391 (18)	0.0226 (16)	0.050 (3)	0.0024 (12)	0.0020 (15)	−0.0048 (16)
C10C	0.0304 (16)	0.0330 (19)	0.037 (2)	0.0017 (12)	−0.0007 (14)	−0.0119 (16)
C11C	0.0239 (14)	0.0292 (17)	0.031 (2)	0.0025 (11)	−0.0002 (12)	−0.0015 (14)
C12C	0.052 (2)	0.0301 (19)	0.070 (3)	−0.0010 (16)	−0.009 (2)	0.0078 (18)
C13C	0.047 (2)	0.052 (2)	0.039 (2)	−0.0212 (17)	0.0042 (17)	0.0021 (17)
O1D	0.0455 (14)	0.0275 (12)	0.0624 (19)	0.0103 (10)	−0.0065 (14)	−0.0039 (12)
N1D	0.0255 (12)	0.0268 (13)	0.0375 (16)	0.0054 (10)	0.0005 (11)	0.0014 (11)
C1D	0.0270 (14)	0.0257 (15)	0.0318 (17)	0.0062 (12)	0.0000 (13)	0.0008 (13)
C2D	0.0287 (14)	0.0236 (14)	0.0397 (19)	0.0005 (11)	−0.0046 (14)	−0.0010 (13)
C3D	0.0384 (18)	0.0251 (15)	0.037 (2)	0.0061 (13)	−0.0032 (15)	−0.0051 (14)
C4D	0.0242 (14)	0.0321 (16)	0.056 (2)	0.0039 (13)	−0.0032 (15)	−0.0058 (16)
C5D	0.0315 (16)	0.0238 (15)	0.037 (2)	−0.0031 (12)	−0.0013 (14)	−0.0021 (14)
C6D	0.0381 (17)	0.0264 (16)	0.0302 (19)	0.0005 (12)	0.0028 (14)	0.0018 (14)
C7D	0.0367 (17)	0.0410 (19)	0.042 (2)	−0.0044 (15)	0.0063 (16)	−0.0023 (16)
C8D	0.061 (2)	0.0346 (18)	0.034 (2)	−0.0183 (16)	0.0113 (18)	−0.0035 (15)
C9D	0.069 (2)	0.0227 (16)	0.0268 (19)	0.0078 (15)	0.0089 (17)	0.0018 (14)
C10D	0.0519 (19)	0.0309 (16)	0.0287 (17)	0.0095 (14)	0.0089 (16)	0.0024 (14)
C11D	0.0378 (17)	0.0271 (15)	0.0263 (16)	0.0022 (12)	0.0028 (14)	0.0045 (13)
C12D	0.0408 (19)	0.046 (2)	0.059 (3)	0.0044 (16)	−0.0154 (18)	−0.0166 (18)
C13D	0.041 (2)	0.0347 (18)	0.064 (3)	−0.0014 (15)	0.0044 (18)	0.0107 (18)

### Geometric parameters (Å, º)

**Table:**

O1A—C3A	1.201 (4)	O1C—C3C	1.206 (4)
N1A—H1A	0.8800	N1C—H1C	0.8800
N1A—C1A	1.366 (4)	N1C—C1C	1.373 (4)
N1A—C11A	1.371 (4)	N1C—C11C	1.390 (5)
C1A—C2A	1.502 (5)	C1C—C2C	1.494 (5)
C1A—C5A	1.350 (5)	C1C—C5C	1.362 (5)
C2A—C3A	1.554 (5)	C2C—C3C	1.554 (5)
C2A—C12A	1.537 (6)	C2C—C12C	1.537 (6)
C2A—C13A	1.531 (5)	C2C—C13C	1.533 (5)
C3A—C4A	1.537 (5)	C3C—C4C	1.534 (5)
C4A—H4AA	0.9900	C4C—H4CA	0.9900
C4A—H4AB	0.9900	C4C—H4CB	0.9900
C4A—C5A	1.498 (4)	C4C—C5C	1.497 (5)
C5A—C6A	1.429 (4)	C5C—C6C	1.428 (4)
C6A—C7A	1.400 (5)	C6C—C7C	1.403 (5)
C6A—C11A	1.424 (5)	C6C—C11C	1.420 (5)
C7A—H7A	0.9500	C7C—H7C	0.9500
C7A—C8A	1.381 (5)	C7C—C8C	1.386 (5)
C8A—H8A	0.9500	C8C—H8C	0.9500
C8A—C9A	1.398 (7)	C8C—C9C	1.395 (6)
C9A—H9A	0.9500	C9C—H9C	0.9500
C9A—C10A	1.378 (6)	C9C—C10C	1.381 (6)
C10A—H10A	0.9500	C10C—H10C	0.9500
C10A—C11A	1.401 (5)	C10C—C11C	1.383 (5)
C12A—H12A	0.9800	C12C—H12G	0.9800
C12A—H12B	0.9800	C12C—H12H	0.9800
C12A—H12C	0.9800	C12C—H12I	0.9800
C13A—H13A	0.9800	C13C—H13G	0.9800
C13A—H13B	0.9800	C13C—H13H	0.9800
C13A—H13C	0.9800	C13C—H13I	0.9800
O1B—C3B	1.204 (4)	O1D—C3D	1.205 (4)
N1B—H1B	0.8800	N1D—H1D	0.8800
N1B—C1B	1.370 (4)	N1D—C1D	1.375 (4)
N1B—C11B	1.379 (4)	N1D—C11D	1.378 (4)
C1B—C2B	1.494 (4)	C1D—C2D	1.486 (4)
C1B—C5B	1.349 (4)	C1D—C5D	1.359 (5)
C2B—C3B	1.561 (4)	C2D—C3D	1.558 (4)
C2B—C12B	1.536 (5)	C2D—C12D	1.529 (5)
C2B—C13B	1.534 (5)	C2D—C13D	1.521 (6)
C3B—C4B	1.523 (4)	C3D—C4D	1.522 (5)
C4B—H4BA	0.9900	C4D—H4DA	0.9900
C4B—H4BB	0.9900	C4D—H4DB	0.9900
C4B—C5B	1.500 (4)	C4D—C5D	1.495 (4)
C5B—C6B	1.437 (4)	C5D—C6D	1.431 (5)
C6B—C7B	1.405 (5)	C6D—C7D	1.409 (5)
C6B—C11B	1.422 (5)	C6D—C11D	1.417 (5)
C7B—H7B	0.9500	C7D—H7D	0.9500
C7B—C8B	1.397 (5)	C7D—C8D	1.391 (5)
C8B—H8B	0.9500	C8D—H8D	0.9500
C8B—C9B	1.391 (5)	C8D—C9D	1.393 (6)
C9B—H9B	0.9500	C9D—H9D	0.9500
C9B—C10B	1.370 (5)	C9D—C10D	1.372 (5)
C10B—H10B	0.9500	C10D—H10D	0.9500
C10B—C11B	1.398 (4)	C10D—C11D	1.390 (5)
C12B—H12D	0.9800	C12D—H12J	0.9800
C12B—H12E	0.9800	C12D—H12K	0.9800
C12B—H12F	0.9800	C12D—H12L	0.9800
C13B—H13D	0.9800	C13D—H13J	0.9800
C13B—H13E	0.9800	C13D—H13K	0.9800
C13B—H13F	0.9800	C13D—H13L	0.9800
			
C1A—N1A—H1A	126.1	C1C—N1C—H1C	126.2
C1A—N1A—C11A	107.8 (3)	C1C—N1C—C11C	107.7 (3)
C11A—N1A—H1A	126.1	C11C—N1C—H1C	126.2
N1A—C1A—C2A	133.5 (3)	N1C—C1C—C2C	133.3 (3)
C5A—C1A—N1A	110.9 (3)	C5C—C1C—N1C	110.9 (3)
C5A—C1A—C2A	115.5 (3)	C5C—C1C—C2C	115.8 (3)
C1A—C2A—C3A	99.1 (3)	C1C—C2C—C3C	99.0 (3)
C1A—C2A—C12A	113.6 (3)	C1C—C2C—C12C	113.5 (3)
C1A—C2A—C13A	113.1 (3)	C1C—C2C—C13C	113.8 (3)
C12A—C2A—C3A	110.3 (4)	C12C—C2C—C3C	109.6 (3)
C13A—C2A—C3A	109.6 (3)	C13C—C2C—C3C	110.0 (3)
C13A—C2A—C12A	110.7 (3)	C13C—C2C—C12C	110.4 (3)
O1A—C3A—C2A	122.7 (4)	O1C—C3C—C2C	122.7 (4)
O1A—C3A—C4A	125.5 (4)	O1C—C3C—C4C	125.2 (4)
C4A—C3A—C2A	111.7 (3)	C4C—C3C—C2C	112.0 (3)
C3A—C4A—H4AA	111.5	C3C—C4C—H4CA	111.4
C3A—C4A—H4AB	111.5	C3C—C4C—H4CB	111.4
H4AA—C4A—H4AB	109.3	H4CA—C4C—H4CB	109.3
C5A—C4A—C3A	101.6 (3)	C5C—C4C—C3C	101.6 (3)
C5A—C4A—H4AA	111.5	C5C—C4C—H4CA	111.4
C5A—C4A—H4AB	111.5	C5C—C4C—H4CB	111.4
C1A—C5A—C4A	112.0 (3)	C1C—C5C—C4C	111.5 (3)
C1A—C5A—C6A	107.6 (3)	C1C—C5C—C6C	107.1 (3)
C6A—C5A—C4A	140.4 (3)	C6C—C5C—C4C	141.4 (3)
C7A—C6A—C5A	135.2 (3)	C7C—C6C—C5C	135.0 (3)
C7A—C6A—C11A	119.6 (3)	C7C—C6C—C11C	118.5 (3)
C11A—C6A—C5A	105.2 (3)	C11C—C6C—C5C	106.4 (3)
C6A—C7A—H7A	120.7	C6C—C7C—H7C	120.3
C8A—C7A—C6A	118.6 (4)	C8C—C7C—C6C	119.3 (4)
C8A—C7A—H7A	120.7	C8C—C7C—H7C	120.3
C7A—C8A—H8A	119.4	C7C—C8C—H8C	120.0
C7A—C8A—C9A	121.3 (4)	C7C—C8C—C9C	120.1 (4)
C9A—C8A—H8A	119.4	C9C—C8C—H8C	120.0
C8A—C9A—H9A	119.1	C8C—C9C—H9C	118.7
C10A—C9A—C8A	121.7 (3)	C10C—C9C—C8C	122.6 (3)
C10A—C9A—H9A	119.1	C10C—C9C—H9C	118.7
C9A—C10A—H10A	121.2	C9C—C10C—H10C	121.5
C9A—C10A—C11A	117.6 (4)	C9C—C10C—C11C	117.0 (4)
C11A—C10A—H10A	121.2	C11C—C10C—H10C	121.5
N1A—C11A—C6A	108.6 (3)	N1C—C11C—C6C	107.9 (3)
N1A—C11A—C10A	130.2 (3)	C10C—C11C—N1C	129.6 (4)
C10A—C11A—C6A	121.2 (3)	C10C—C11C—C6C	122.5 (3)
C2A—C12A—H12A	109.5	C2C—C12C—H12G	109.5
C2A—C12A—H12B	109.5	C2C—C12C—H12H	109.5
C2A—C12A—H12C	109.5	C2C—C12C—H12I	109.5
H12A—C12A—H12B	109.5	H12G—C12C—H12H	109.5
H12A—C12A—H12C	109.5	H12G—C12C—H12I	109.5
H12B—C12A—H12C	109.5	H12H—C12C—H12I	109.5
C2A—C13A—H13A	109.5	C2C—C13C—H13G	109.5
C2A—C13A—H13B	109.5	C2C—C13C—H13H	109.5
C2A—C13A—H13C	109.5	C2C—C13C—H13I	109.5
H13A—C13A—H13B	109.5	H13G—C13C—H13H	109.5
H13A—C13A—H13C	109.5	H13G—C13C—H13I	109.5
H13B—C13A—H13C	109.5	H13H—C13C—H13I	109.5
C1B—N1B—H1B	126.2	C1D—N1D—H1D	126.1
C1B—N1B—C11B	107.7 (3)	C1D—N1D—C11D	107.9 (3)
C11B—N1B—H1B	126.2	C11D—N1D—H1D	126.1
N1B—C1B—C2B	133.3 (3)	N1D—C1D—C2D	133.7 (3)
C5B—C1B—N1B	111.2 (3)	C5D—C1D—N1D	110.4 (3)
C5B—C1B—C2B	115.5 (3)	C5D—C1D—C2D	115.8 (3)
C1B—C2B—C3B	99.0 (2)	C1D—C2D—C3D	98.9 (2)
C1B—C2B—C12B	113.2 (3)	C1D—C2D—C12D	113.3 (3)
C1B—C2B—C13B	113.8 (3)	C1D—C2D—C13D	113.4 (3)
C12B—C2B—C3B	110.4 (3)	C12D—C2D—C3D	110.5 (3)
C13B—C2B—C3B	109.4 (3)	C13D—C2D—C3D	109.0 (3)
C13B—C2B—C12B	110.4 (3)	C13D—C2D—C12D	111.2 (3)
O1B—C3B—C2B	123.1 (3)	O1D—C3D—C2D	122.8 (3)
O1B—C3B—C4B	125.2 (3)	O1D—C3D—C4D	125.2 (3)
C4B—C3B—C2B	111.7 (3)	C4D—C3D—C2D	112.0 (3)
C3B—C4B—H4BA	111.5	C3D—C4D—H4DA	111.4
C3B—C4B—H4BB	111.5	C3D—C4D—H4DB	111.4
H4BA—C4B—H4BB	109.3	H4DA—C4D—H4DB	109.3
C5B—C4B—C3B	101.6 (3)	C5D—C4D—C3D	101.7 (3)
C5B—C4B—H4BA	111.5	C5D—C4D—H4DA	111.4
C5B—C4B—H4BB	111.5	C5D—C4D—H4DB	111.4
C1B—C5B—C4B	112.0 (3)	C1D—C5D—C4D	111.4 (3)
C1B—C5B—C6B	107.2 (3)	C1D—C5D—C6D	107.4 (3)
C6B—C5B—C4B	140.8 (3)	C6D—C5D—C4D	141.2 (3)
C7B—C6B—C5B	135.7 (3)	C7D—C6D—C5D	135.1 (3)
C7B—C6B—C11B	118.7 (3)	C7D—C6D—C11D	119.1 (3)
C11B—C6B—C5B	105.6 (3)	C11D—C6D—C5D	105.9 (3)
C6B—C7B—H7B	120.7	C6D—C7D—H7D	121.0
C8B—C7B—C6B	118.6 (3)	C8D—C7D—C6D	118.1 (3)
C8B—C7B—H7B	120.7	C8D—C7D—H7D	121.0
C7B—C8B—H8B	119.5	C7D—C8D—H8D	119.2
C9B—C8B—C7B	120.9 (3)	C7D—C8D—C9D	121.6 (3)
C9B—C8B—H8B	119.5	C9D—C8D—H8D	119.2
C8B—C9B—H9B	118.9	C8D—C9D—H9D	119.4
C10B—C9B—C8B	122.3 (3)	C10D—C9D—C8D	121.2 (3)
C10B—C9B—H9B	118.9	C10D—C9D—H9D	119.4
C9B—C10B—H10B	121.3	C9D—C10D—H10D	120.9
C9B—C10B—C11B	117.4 (3)	C9D—C10D—C11D	118.2 (3)
C11B—C10B—H10B	121.3	C11D—C10D—H10D	120.9
N1B—C11B—C6B	108.3 (3)	N1D—C11D—C6D	108.4 (3)
N1B—C11B—C10B	129.5 (3)	N1D—C11D—C10D	129.8 (3)
C10B—C11B—C6B	122.1 (3)	C10D—C11D—C6D	121.8 (3)
C2B—C12B—H12D	109.5	C2D—C12D—H12J	109.5
C2B—C12B—H12E	109.5	C2D—C12D—H12K	109.5
C2B—C12B—H12F	109.5	C2D—C12D—H12L	109.5
H12D—C12B—H12E	109.5	H12J—C12D—H12K	109.5
H12D—C12B—H12F	109.5	H12J—C12D—H12L	109.5
H12E—C12B—H12F	109.5	H12K—C12D—H12L	109.5
C2B—C13B—H13D	109.5	C2D—C13D—H13J	109.5
C2B—C13B—H13E	109.5	C2D—C13D—H13K	109.5
C2B—C13B—H13F	109.5	C2D—C13D—H13L	109.5
H13D—C13B—H13E	109.5	H13J—C13D—H13K	109.5
H13D—C13B—H13F	109.5	H13J—C13D—H13L	109.5
H13E—C13B—H13F	109.5	H13K—C13D—H13L	109.5
			
O1A—C3A—C4A—C5A	−177.7 (4)	O1C—C3C—C4C—C5C	176.0 (4)
N1A—C1A—C2A—C3A	177.7 (4)	N1C—C1C—C2C—C3C	−179.4 (4)
N1A—C1A—C2A—C12A	−65.4 (5)	N1C—C1C—C2C—C12C	64.6 (5)
N1A—C1A—C2A—C13A	61.8 (5)	N1C—C1C—C2C—C13C	−62.7 (5)
N1A—C1A—C5A—C4A	−176.8 (3)	N1C—C1C—C5C—C4C	178.2 (3)
N1A—C1A—C5A—C6A	0.4 (4)	N1C—C1C—C5C—C6C	−0.8 (4)
C1A—N1A—C11A—C6A	−0.6 (4)	C1C—N1C—C11C—C6C	0.0 (4)
C1A—N1A—C11A—C10A	178.3 (3)	C1C—N1C—C11C—C10C	−178.5 (3)
C1A—C2A—C3A—O1A	178.3 (4)	C1C—C2C—C3C—O1C	−176.9 (4)
C1A—C2A—C3A—C4A	−1.8 (4)	C1C—C2C—C3C—C4C	1.1 (4)
C1A—C5A—C6A—C7A	−179.2 (4)	C1C—C5C—C6C—C7C	179.9 (4)
C1A—C5A—C6A—C11A	−0.8 (3)	C1C—C5C—C6C—C11C	0.7 (3)
C2A—C1A—C5A—C4A	1.0 (4)	C2C—C1C—C5C—C4C	−1.5 (4)
C2A—C1A—C5A—C6A	178.3 (3)	C2C—C1C—C5C—C6C	179.5 (3)
C2A—C3A—C4A—C5A	2.3 (4)	C2C—C3C—C4C—C5C	−1.9 (4)
C3A—C4A—C5A—C1A	−2.0 (4)	C3C—C4C—C5C—C1C	2.1 (4)
C3A—C4A—C5A—C6A	−177.9 (4)	C3C—C4C—C5C—C6C	−179.5 (4)
C4A—C5A—C6A—C7A	−3.1 (7)	C4C—C5C—C6C—C7C	1.4 (7)
C4A—C5A—C6A—C11A	175.2 (4)	C4C—C5C—C6C—C11C	−177.7 (4)
C5A—C1A—C2A—C3A	0.5 (4)	C5C—C1C—C2C—C3C	0.2 (4)
C5A—C1A—C2A—C12A	117.4 (4)	C5C—C1C—C2C—C12C	−115.8 (4)
C5A—C1A—C2A—C13A	−115.4 (4)	C5C—C1C—C2C—C13C	116.9 (4)
C5A—C6A—C7A—C8A	177.2 (3)	C5C—C6C—C7C—C8C	−179.1 (3)
C5A—C6A—C11A—N1A	0.8 (3)	C5C—C6C—C11C—N1C	−0.4 (3)
C5A—C6A—C11A—C10A	−178.2 (3)	C5C—C6C—C11C—C10C	178.1 (3)
C6A—C7A—C8A—C9A	0.7 (5)	C6C—C7C—C8C—C9C	0.7 (5)
C7A—C6A—C11A—N1A	179.5 (3)	C7C—C6C—C11C—N1C	−179.8 (3)
C7A—C6A—C11A—C10A	0.5 (5)	C7C—C6C—C11C—C10C	−1.2 (5)
C7A—C8A—C9A—C10A	0.3 (5)	C7C—C8C—C9C—C10C	−0.2 (6)
C8A—C9A—C10A—C11A	−0.9 (5)	C8C—C9C—C10C—C11C	−0.9 (5)
C9A—C10A—C11A—N1A	−178.3 (3)	C9C—C10C—C11C—N1C	179.8 (3)
C9A—C10A—C11A—C6A	0.5 (5)	C9C—C10C—C11C—C6C	1.6 (5)
C11A—N1A—C1A—C2A	−177.2 (4)	C11C—N1C—C1C—C2C	−179.8 (3)
C11A—N1A—C1A—C5A	0.1 (4)	C11C—N1C—C1C—C5C	0.5 (4)
C11A—C6A—C7A—C8A	−1.1 (5)	C11C—C6C—C7C—C8C	0.0 (5)
C12A—C2A—C3A—O1A	58.9 (5)	C12C—C2C—C3C—O1C	−57.9 (5)
C12A—C2A—C3A—C4A	−121.2 (3)	C12C—C2C—C3C—C4C	120.1 (3)
C13A—C2A—C3A—O1A	−63.2 (5)	C13C—C2C—C3C—O1C	63.6 (5)
C13A—C2A—C3A—C4A	116.8 (4)	C13C—C2C—C3C—C4C	−118.4 (3)
O1B—C3B—C4B—C5B	174.0 (4)	O1D—C3D—C4D—C5D	175.3 (4)
N1B—C1B—C2B—C3B	177.2 (4)	N1D—C1D—C2D—C3D	178.6 (4)
N1B—C1B—C2B—C12B	−66.0 (5)	N1D—C1D—C2D—C12D	−64.4 (5)
N1B—C1B—C2B—C13B	61.2 (5)	N1D—C1D—C2D—C13D	63.4 (5)
N1B—C1B—C5B—C4B	179.8 (3)	N1D—C1D—C5D—C4D	178.9 (3)
N1B—C1B—C5B—C6B	0.0 (4)	N1D—C1D—C5D—C6D	0.0 (4)
C1B—N1B—C11B—C6B	0.6 (4)	C1D—N1D—C11D—C6D	−0.1 (4)
C1B—N1B—C11B—C10B	−177.7 (4)	C1D—N1D—C11D—C10D	−179.2 (4)
C1B—C2B—C3B—O1B	−174.5 (4)	C1D—C2D—C3D—O1D	−176.7 (4)
C1B—C2B—C3B—C4B	4.4 (4)	C1D—C2D—C3D—C4D	1.5 (4)
C1B—C5B—C6B—C7B	178.4 (4)	C1D—C5D—C6D—C7D	179.6 (4)
C1B—C5B—C6B—C11B	0.3 (4)	C1D—C5D—C6D—C11D	0.0 (4)
C2B—C1B—C5B—C4B	−0.6 (5)	C2D—C1D—C5D—C4D	−2.7 (5)
C2B—C1B—C5B—C6B	179.6 (3)	C2D—C1D—C5D—C6D	178.4 (3)
C2B—C3B—C4B—C5B	−4.8 (4)	C2D—C3D—C4D—C5D	−2.9 (4)
C3B—C4B—C5B—C1B	3.3 (4)	C3D—C4D—C5D—C1D	3.3 (4)
C3B—C4B—C5B—C6B	−177.1 (5)	C3D—C4D—C5D—C6D	−178.3 (5)
C4B—C5B—C6B—C7B	−1.2 (8)	C4D—C5D—C6D—C7D	1.2 (9)
C4B—C5B—C6B—C11B	−179.3 (5)	C4D—C5D—C6D—C11D	−178.5 (5)
C5B—C1B—C2B—C3B	−2.3 (4)	C5D—C1D—C2D—C3D	0.7 (4)
C5B—C1B—C2B—C12B	114.6 (4)	C5D—C1D—C2D—C12D	117.6 (4)
C5B—C1B—C2B—C13B	−118.3 (4)	C5D—C1D—C2D—C13D	−114.5 (4)
C5B—C6B—C7B—C8B	−177.4 (4)	C5D—C6D—C7D—C8D	−178.8 (4)
C5B—C6B—C11B—N1B	−0.5 (4)	C5D—C6D—C11D—N1D	0.1 (4)
C5B—C6B—C11B—C10B	177.8 (3)	C5D—C6D—C11D—C10D	179.2 (3)
C6B—C7B—C8B—C9B	0.0 (5)	C6D—C7D—C8D—C9D	−0.5 (6)
C7B—C6B—C11B—N1B	−179.0 (3)	C7D—C6D—C11D—N1D	−179.6 (3)
C7B—C6B—C11B—C10B	−0.6 (5)	C7D—C6D—C11D—C10D	−0.5 (6)
C7B—C8B—C9B—C10B	−0.3 (6)	C7D—C8D—C9D—C10D	−0.3 (6)
C8B—C9B—C10B—C11B	0.2 (6)	C8D—C9D—C10D—C11D	0.7 (6)
C9B—C10B—C11B—N1B	178.3 (4)	C9D—C10D—C11D—N1D	178.7 (4)
C9B—C10B—C11B—C6B	0.3 (5)	C9D—C10D—C11D—C6D	−0.3 (5)
C11B—N1B—C1B—C2B	−179.9 (4)	C11D—N1D—C1D—C2D	−177.9 (4)
C11B—N1B—C1B—C5B	−0.4 (4)	C11D—N1D—C1D—C5D	0.1 (4)
C11B—C6B—C7B—C8B	0.5 (5)	C11D—C6D—C7D—C8D	0.9 (6)
C12B—C2B—C3B—O1B	66.5 (5)	C12D—C2D—C3D—O1D	64.2 (5)
C12B—C2B—C3B—C4B	−114.6 (3)	C12D—C2D—C3D—C4D	−117.5 (4)
C13B—C2B—C3B—O1B	−55.2 (5)	C13D—C2D—C3D—O1D	−58.2 (5)
C13B—C2B—C3B—C4B	123.7 (3)	C13D—C2D—C3D—C4D	120.1 (4)

### Hydrogen-bond geometry (Å, º)

**Table:**

*D*—H···*A*	*D*—H	H···*A*	*D*···*A*	*D*—H···*A*
C9*B*—H9*B*···O1*B*^i^	0.95	2.51	3.427 (4)	162
C10*B*—H10*B*···O1*A*	0.95	2.61	3.379 (5)	139
C12*B*—H12*D*···O1*C*^ii^	0.98	2.73	3.497 (6)	136
C9*D*—H9*D*···O1*D*^iii^	0.95	2.46	3.372 (4)	161
C10*D*—H10*D*···O1*C*^ii^	0.95	2.46	3.274 (5)	144
C12*D*—H12*J*···O1*A*^iv^	0.98	2.69	3.485 (6)	138

Symmetry codes: (i) −*x*+1, *y*+1/2, *z*; (ii) −*x*+1, *y*−1/2, *z*; (iii) −*x*, *y*−1/2, *z*; (iv) *x*−1, *y*, *z*.
